# A pilot study on the importance of forefoot bone length in male 400-m sprinters: is there a key morphological factor for superior long sprint performance?

**DOI:** 10.1186/s13104-018-3685-y

**Published:** 2018-08-13

**Authors:** Daichi Tomita, Tadashi Suga, Takahiro Tanaka, Hiromasa Ueno, Yuto Miyake, Mitsuo Otsuka, Akinori Nagano, Tadao Isaka

**Affiliations:** Faculty of Sport and Health Science, Ritsumeikan University, 1-1-1 Nojihigashi, Kusatsu, Shiga 525-8577 Japan

**Keywords:** Sprint velocity, Plantar flexor moment, Ground reaction force, Anaerobic capacity, Magnetic resonance imaging

## Abstract

**Objective:**

The main purpose of the present study was to determine the relationship between the forefoot bone length and long sprint performance in well-trained 400-m specialized sprinters. The total lengths of the forefoot bones of the big and second toes in 25 male 400-m sprinters and 25 male non-sprinters were measured using magnetic resonance imaging. The forefoot bones of each toe were totaled to assess overall forefoot bone length and then normalized to the maximum foot length.

**Results:**

The relative total lengths of the forefoot bones in the big and second toes were significantly longer in 400-m sprinters than in non-sprinters (*P* < 0.05 for both). The relative total length of the forefoot bones of the second toe, but not of the big toe, in 400-m sprinters was significantly correlated with personal best 400-m sprint time (*r* = − 0.441, *P* = 0.028). These findings demonstrated that longer forefoot bones are related to higher long sprint performance in well-trained 400-m specialized sprinters. Therefore, the present study is the first to determine that morphological factors such as long forefoot bones may play an important role in achieving superior long sprinting performance.

## Introduction

In a previous study, Lee and Piazza [2] first reported that the forefoot was longer in sprinters than in non-sprinters. Thereafter, using magnetic resonance imaging (MRI), Baxter et al. [1] demonstrated that the forefoot bone length of the big toe was longer in sprinters than in non-sprinters. Our previous study, which had a relatively large sample (n = 32 per group), supported their findings by showing that MRI-measured lengths of the forefoot bones of the big and second toes were longer in sprinters than in non-sprinters [3]. Therefore, these findings suggest that sprinters have a unique forefoot structure with longer bone length than non-sprinters.

As revealed using computer simulation, Lee and Piazza [2] showed that a longer forefoot may contribute to higher sprint performance by potentially enhancing plantar flexor moment during the push-off phase of the stance phase; however, this relationship has not been directly demonstrated in humans. In a recent study, we demonstrated that longer forefoot bones are related to higher 100-m sprint performance in sprinters [3]. Therefore, the forefoot bone length may be a key morphological factor in achieving superior sprint performance because of enhanced plantar flexor moment.

Although previous studies have determined positive relationships between some morphological factors and 100-m sprint performance [3–8], to the best of our knowledge, such beneficial morphological factors for long sprint performances (e.g., 400-m sprint) have not been identified. This may be due to the evidence that superior 400-m sprint performance is mainly determined by physiological factors, such as anaerobic capacity and fatigue resistance [9–12]. However, Morin et al. [13] reported that greater horizontal and vertical reaction forces are related to higher maximal sprint velocity during sprinting. Additionally, Hobara et al. [14] determined that the decrease in sprint velocity during 400-m sprinting is in parallel with that in vertical ground reaction force. As is well known, the magnitudes of these reaction forces are mediated by the plantar flexor moment [15]. Considering these findings, longer forefoot bones may help achieve higher long sprint performance, potentially by improving the ability to attain maximal sprint velocity, to maintain its velocity, or both, which may be due to enhancements in the plantar flexor moment and horizontal and vertical ground reaction forces. Thus, we hypothesized that a favorable forefoot morphology would be related to superior long sprint performance. To test our hypothesis, in this pilot study, we first compared the lengths of the forefoot bones in 400-m sprinters and non-sprinters to gain a better understanding of the characteristics of the forefoot bones in 400-m sprinters. Thereafter, we examined the relationship between the forefoot bone length and long sprint performance in 400-m sprinters.

## Main text

### Methods

Twenty-five well-trained male sprinters (age 20.2 ± 1.7 years) participated in this study. They specialized in 400-m race, and their personal best 400-m sprint times ranged from 46.07 to 51.90 s (mean 49.23 ± 1.46 s). Additionally, 25 male non-sprinters (age 21.4 ± 2.1 years) matched to the sprinters for body height and body weight participated as a control group. None of the participants had contraindications to MRI. All participants were informed of the experimental procedures and provided written consent to participate in the study. All procedures were approved by the Ethics Committee of Ritsumeikan University (BKC-IRB-2011-009).

For anthropometric data, the length of the right foot of participants was measured in millimeters as the distance from the heel to the end of the big and second toes. The highest value in both toes was considered the maximum foot length, and this length was used to normalize bone length [3].

**Fig. 1:**
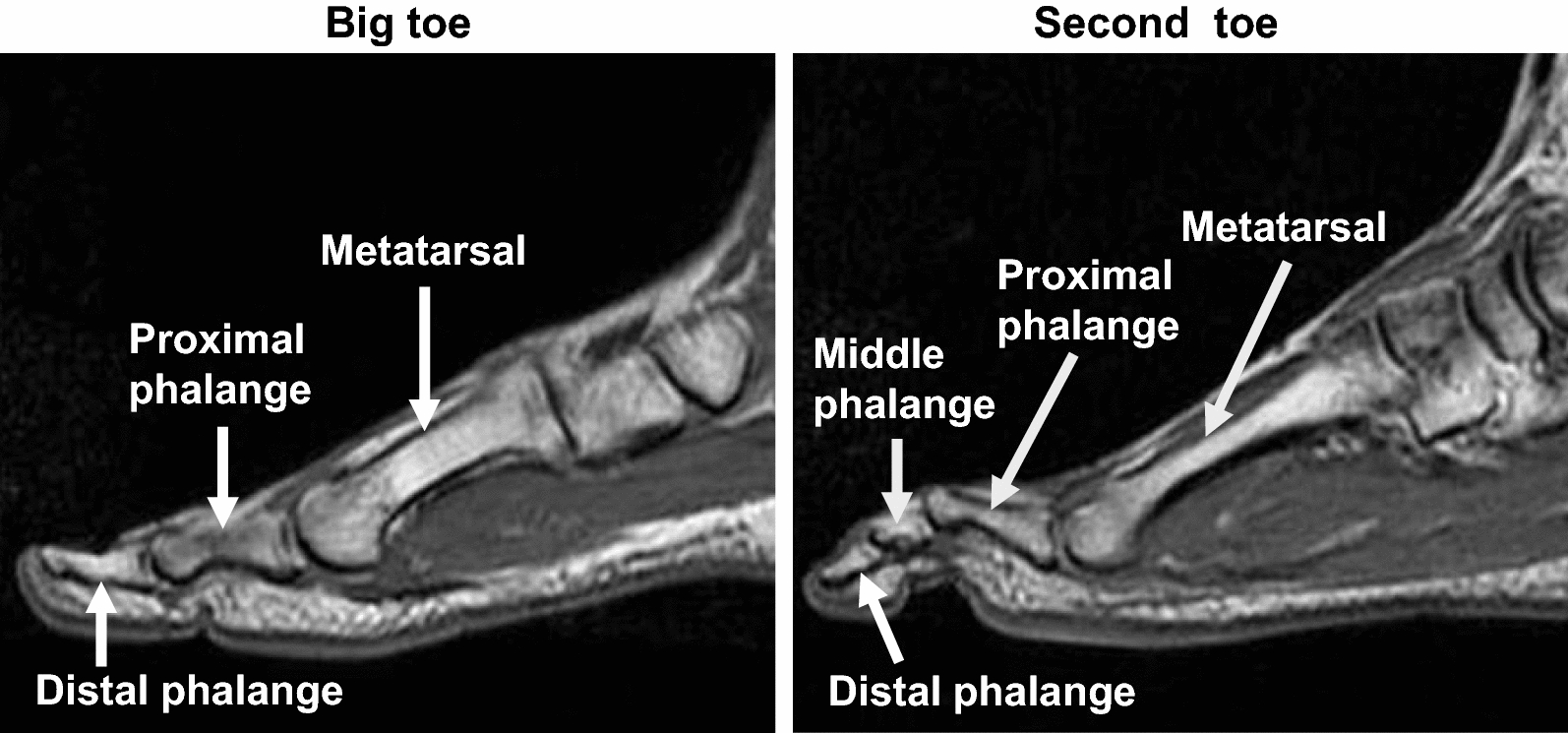
Representative magnetic resonance imaging scans used for measuring the lengths of the forefoot bones of the big and second toes. The forefoot bones of the big toe included the distal phalanx, proximal phalanx, and metatarsal bones. The forefoot bones of the second toe included the distal phalanx, middle phalanx, proximal phalanx, and metatarsal bones

Representative MRI scans used for measuring the lengths of the forefoot bones of the big and second toes are shown in Fig. 1. The bone lengths of the right foot of participants were measured using a 1.5-T magnetic resonance system (Signa HDxt; GE Medical Systems, USA). The methods for performing MRI scans and measuring the lengths of foot bones have been previously described [3]. Each bone length was measured twice by a single examiner, and the two values were averaged. The intraclass correlation coefficient of the two measurements for each bone length ranged from 0.937 to 0.994. These analyses were conducted using image analysis software (OsiriX Version 5.6; OsiriX Foundation, Switzerland).

Data are presented as the mean ± SD. Comparisons of groups were performed using an unpaired *t* test. The relationship between forefoot bone length and personal best 400-m sprint time in 400-m sprinters was examined using the Pearson’s product-moment correlation coefficient. The strength of the correlation coefficient was assessed using Evans’ classification [16]. Moreover, partial correlation analyses were performed after controlling for confounding factors to examine the relationship between forefoot bone length and personal best 400-m sprint time. The model was adjusted for body height and body weight. Furthermore, a stepwise multiple regression analysis was used to determine the predictive variable(s) for the personal best 400-m sprint time using the absolute and relative lengths of selected bones (i.e., total forefoot bones of big and second toes, mid-foot bones, and rearfoot bones) as independent variables. The statistical significance level was defined at *P* < 0.05. All statistical analyses were conducted using IBM SPSS software (version 19.0; International Business Machines Corp, USA).

### Results

Physical characteristics and foot morphologies in 400-m sprinters and non-sprinters are listed in Table 1. Body height and body weight did not differ significantly between 400-m sprinters and non-sprinters. The foot length also did not differ significantly between the two groups.

**Table 1:** Physical characteristics and foot morphologies in 400-m sprinters and non-sprinters

	Sprinters	Non-sprinters	*P* value
Body height, cm	175.4 ± 5.1	174.4 ± 4.2	0.452
Body weight, kg	64.8 ± 5.1	66.3 ± 6.1	0.358
Heel to big toe, cm	26.2 ± 0.7	26.1 ± 1.1	0.809
Heel to second toe, cm	25.9 ± 0.9	25.9 ± 1.0	0.988
Maximum foot length, cm	26.2 ± 0.7	26.2 ± 1.1	0.879
Forefoot bones of the big toe			
Distal phalanx, mm	23.5 ± 1.7	23.9 ± 1.9	0.474
Proximal phalanx, mm	33.4 ± 1.8	32.5 ± 2.0	0.117
Metatarsal, mm	*66.9 ± 2.5*	*64.2 ± 3.3*	*0.002*
Total length, mm	*123.8 ± 5.1*	*120.6 ± 5.1*	*0.030*
Relative total length, % of FL	*47.2 ± 1.6*	*46.0 ± 1.1*	*0.006*
Forefoot bones of the second toe			
Distal phalanx, mm	11.1 ± 1.2	11.1 ± 1.2	0.962
Middle phalanx, mm	13.0 ± 2.2	12.2 ± 2.3	0.209
Proximal phalanx, mm	30.9 ± 1.9	30.2 ± 1.4	0.114
Metatarsal, mm	*81.0 ± 2.3*	*79.5 ± 3.0*	*0.044*
Total length, mm	*136.2 ± 5.2*	*133.0 ± 5.2*	*0.038*
Relative total length, % of FL	*51.9 ± 1.4*	*50.8 ± 1.5*	*0.010*
Mid-foot bones (mm)			
Medial cuneiform	24.8 ± 1.7	24.8 ± 1.7	0.993
Intermediate cuneiform	20.5 ± 1.2	20.8 ± 1.3	0.349
Navicular	19.7 ± 1.9	19.8 ± 1.6	0.692
Rearfoot bones (mm)			
Talus	59.8 ± 2.6	60.7 ± 2.7	0.221
Calcaneus	79.8 ± 2.8	81.3 ± 3.5	0.100

Values are presented as mean ± SD. The relative total lengths of the forefoot bones of the big and second toes were normalized with the maximum foot length (FL), and expressed as a percentage. Italics values indicate a significant difference (*P* < 0.05) between sprinters and non-sprinters

Absolute total lengths of the forefoot bones in the big and second toes were significantly longer in 400-m sprinters than in non-sprinters. After normalization with the maximum foot length, the relative total lengths of the forefoot bones in the big and second toes were also significantly longer in 400-m sprinters than in non-sprinters. In contrast, the absolute and relative mid-foot bones did not differ significantly between 400-m sprinters and non-sprinters. Similarly, the absolute and relative rearfoot bones did not differ significantly between the two groups.

**Fig. 2:**
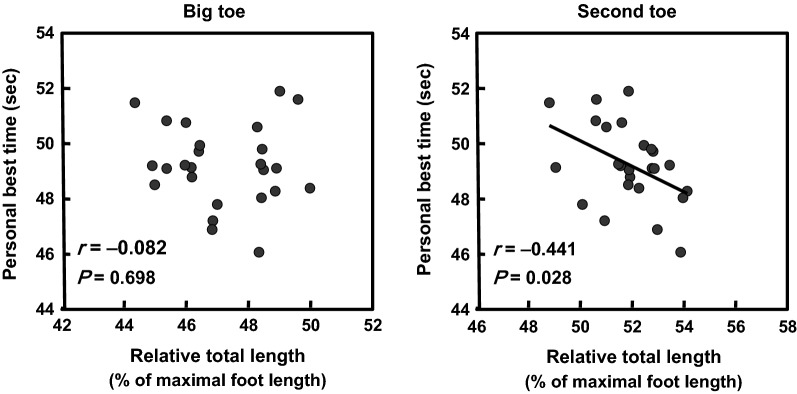
Relationships between the relative total lengths of the forefoot bones of the big and second toes and personal best 400-m sprint time in 400-m sprinters

The absolute total length of the forefoot bones of the second toe, but not of the big toe, in 400-m sprinters correlated significantly with personal best 400-m sprint time, with a moderate correlation (*r* = − 0.411, *R*^2^ = 0.169, *P* = 0.041). Moreover, the relative total length of the forefoot bones of the second toe in 400-m sprinters correlated significantly with personal best 400-m sprint times, with a moderate correlation (*r *= − 0.441, *R*^2^ = 0.194, *P* = 0.028; Fig. 2). After adjusting for body height and body weight, correlation between the relative forefoot bone length and personal best 400-m sprint time remained significant (partial *r* = − 0.540, *P* = 0.008). Furthermore, using a stepwise multiple regression analysis, the following prediction equation of personal best 400-m sprint time was determined for the absolute and relative bone lengths: Personal best 400-m sprint time = (− 0.557) (relative forefoot bone length of second toe) + (− 0.198) (absolute calcaneus length) + 94.001 (*R*^2^ = 0.271, *P* = 0.012). The relative forefoot bone length of second toe was most predictive variable of personal best 400-m sprint time (*β* = − 0.536, *P* = 0.007).

### Discussion

In the present study, we demonstrated that the absolute and relative total lengths of the forefoot bones of the big and second toes were longer in 400-m sprinters than in non-sprinters. As expected, we further demonstrated that the relative total length of the forefoot bones of the second toe was correlated with long sprint performance in 400-m sprinters. Moreover, after adjusting for body height and body weight, a correlation remained significant. Furthermore, using a stepwise multiple regression analysis, we demonstrated that the relative total length of the forefoot bones of the second toe was an important variable for predicting long sprint performance. Therefore, these present findings suggest that longer forefoot bones may be advantageous for higher long sprint performance in 400-m sprinters.

As is well known, the maximal sprint velocity during 400-m sprinting is lower than that during 100-m sprinting [17]. However, Hanon and Gajar [18] reported that the maximal velocity between 50 m and 100 m during 400-m sprinting equated to more than 10 m/s (while performing 400-m sprinting for 44.43 s) for a world-crass sprinter competing in a 400-m race. Moreover, their findings showed that the maximal velocity of this section equated to more than 9 m/s for an elite 400-m sprinter while performing 400-m sprinting for 48.24 s [18], which is similar to or slower than the personal best 400-m sprint time in some sprinters who participated in the present study. Naturally, the ability to attain maximal velocity is required for achieving superior long sprint performance in 400-m sprinters [18]. In a previous study, Lee and Piazza [2] used computer simulation to demonstrate that increased forefoot length can be enhanced by plantar flexor moment during the push-off phase of the stance phase while sprinting. The enhanced plantar flexor moment induced by long forefoot bones may contribute to achieving superior sprint performance, potentially by increasing horizontal and vertical reaction forces, which are important kinetic factors for achieving superior sprint performance [13]. Therefore, longer forefoot bones may be useful in attaining maximal sprint velocity during the early phase of 400-m sprinting.

In addition to the ability to attain maximal sprint velocity, the ability to maintain maximal sprint velocity is required for achieving superior long sprint performance in 400-m sprinters [9, 17, 18]. Indeed, a decrease in sprint velocity during the late phase of 400-m sprinting is connected to the performance outcome of 400-m sprinting [18]. Most previous studies have determined that the ability to maintain maximal sprint velocity in the late phase of 400-m sprinting is dependent on physiological factors (e.g., anaerobic capacity and fatigue resistance [9–12]. However, Hobara et al. [14] reported that the decrease in sprint velocity during 400-m sprinting is parallel with that in vertical ground reaction force, which is mediated by the plantar flexor moment. Therefore, longer forefoot bone may be useful in mitigating the decrease in sprint velocity, potentially by ensuring the required plantar flexor moment for superior sprint performance.

In conclusion, the present study demonstrated that longer forefoot bones are related to higher long sprint performance in well-trained 400-m specialized sprinters. For first to the best of our knowledge, in this pilot study, we could provide evidence that, in addition to physiological factors, morphological factors such as long forefoot bones may play an important role in superior long sprinting performance. In clinical perspectives, information from the present study may help in understanding individual features and selecting the elite in junior athletes. Additionally, our findings indicate that although splinters having longer forefoot bones may have advantages for enhancing favorable kinetic variables and achieving superior sprint performance, none of these can be obtained in sprinters having shorter forefoot bones. Therefore, information from the present study can be further utilized in outlining an optimal training/rehabilitation program and improving outcomes in adult sprinters.

## Limitations

The present study has several limitations. First, because the lengths of midfoot and rearfoot bones did not differ between sprinters and non-sprinters, we could not provide exact data (e.g., arch height) to support the present findings that the forefoot bone lengths of the big and second toes were longer in sprinters than in non-sprinters. Next, we hypothesized that longer forefoot bones may contribute to higher long sprint performance by attaining and/or maintaining maximal velocity in 400-m sprinters. Additionally, we hypothesized that longer forefoot bones may contribute to enhancing plantar flexor moment and horizontal and vertical ground reaction forces during the early phase and to mitigating the reduction in these kinetic variables during the late phase of 400-m sprinting. To test our hypotheses, further studies are needed to examine the relationships between forefoot bone length and race data and kinetic variables during 400-m sprinting.

## Abbreviation

MRI: magnetic resonance imaging
